# Case Report: Extensive deep vein thrombosis and venous pseudoaneurysm following percutaneous transcatheter tricuspid valve intervention

**DOI:** 10.3389/fcvm.2025.1568102

**Published:** 2025-05-16

**Authors:** Zaid Abood, M. Fuad Jan, Eric S. Weiss, Tanvir Bajwa, Suhail Q. Allaqaband, Mark W. Mewissen

**Affiliations:** ^1^ Aurora Cardiovascular and Thoracic Services, Aurora Sinai/Aurora St. Luke’s Medical Centers, Aurora Health Care, Milwaukee, WI, United States; ^2^ Division of Cardiovascular Medicine, University of Wisconsin School of Medicine and Public Health, Milwaukee Clinical Campus, Milwaukee, WI, United States

**Keywords:** access site complication, case report, deep vein thrombosis, phlegmasia cerulea dolens, transcatheter tricuspid valve replacement, venous pseudoaneurysm

## Abstract

**Introduction:**

Transcatheter tricuspid valve intervention (TTVI) has evolved as a less-invasive alternative to surgical treatment of severe tricuspid regurgitation. Although venously delivered valves have been introduced, the risk of venous access site complications is unknown. We present a patient who suffered phlegmasia cerulea dolens post-TTVI.

**Case summary:**

We present an 88-year-old female patient who developed phlegmasia cerulea dolens of the right lower extremity shortly after successful TTVI delivered through the right common femoral vein via a 35F sheath. Ipsilateral transpopliteal venography demonstrated an occlusive thrombus in the right common femoral vein and the incidental finding of an external iliac vein pseudoaneurysm. Endovascular treatment consisting of mechanical thrombectomy followed by adjunctive placement of self-expanding metallic stents resulted in restoration of iliofemoral venous outflow and excellent recovery with resolution of the patient's symptoms.

**Conclusions:**

Surveillance, e.g., duplex ultrasonography, immediately post-TTVI is important to rule out acute thrombosis of the venous access site and other complications associated with a large sheath.

## 1 Introduction

Transcatheter tricuspid valve intervention (TTVI) has evolved as a less-invasive alternative to surgical treatment of severe tricuspid regurgitation (TR). Venously delivered valves with different sheath sizes have been introduced (1). However, the risk of venous access site complications is unknown. We present a patient who experienced phlegmasia cerulea dolens post-TTVI.

## 2 Case description

An 88-year-old woman with stage 3 chronic kidney disease and severe TR underwent elective TTVI with a 48 mm valve delivered via a 35F sheath through the right common femoral vein (CFV). The pre-procedural imaging evaluation, performed while the patient was in a euvolemic, compensated state and on a stable diuretic dose, included transthoracic and transesophageal echocardiography and detailed cardiac computed tomography. The computed tomographic evaluation in this case specifically demonstrated normal anatomy and patency of the inferior vena cava and pelvic veins. Ultrasound-guided access and preclose technique were used. After successful valve deployment, the CFV was sealed with the Perclose ProStyle Suture-Mediated Closure and Repair System (Abbott Cardiovascular, Abbott Park, Ill.). Total fluoroscopy time was 71 min. Intravenous heparin was administered intra-procedurally, with a target activated clotting time >250 s maintained throughout. Heparin reversal with protamine was deemed unnecessary. Post-procedure, antiplatelet therapy was not prescribed.

**Figure 1:**
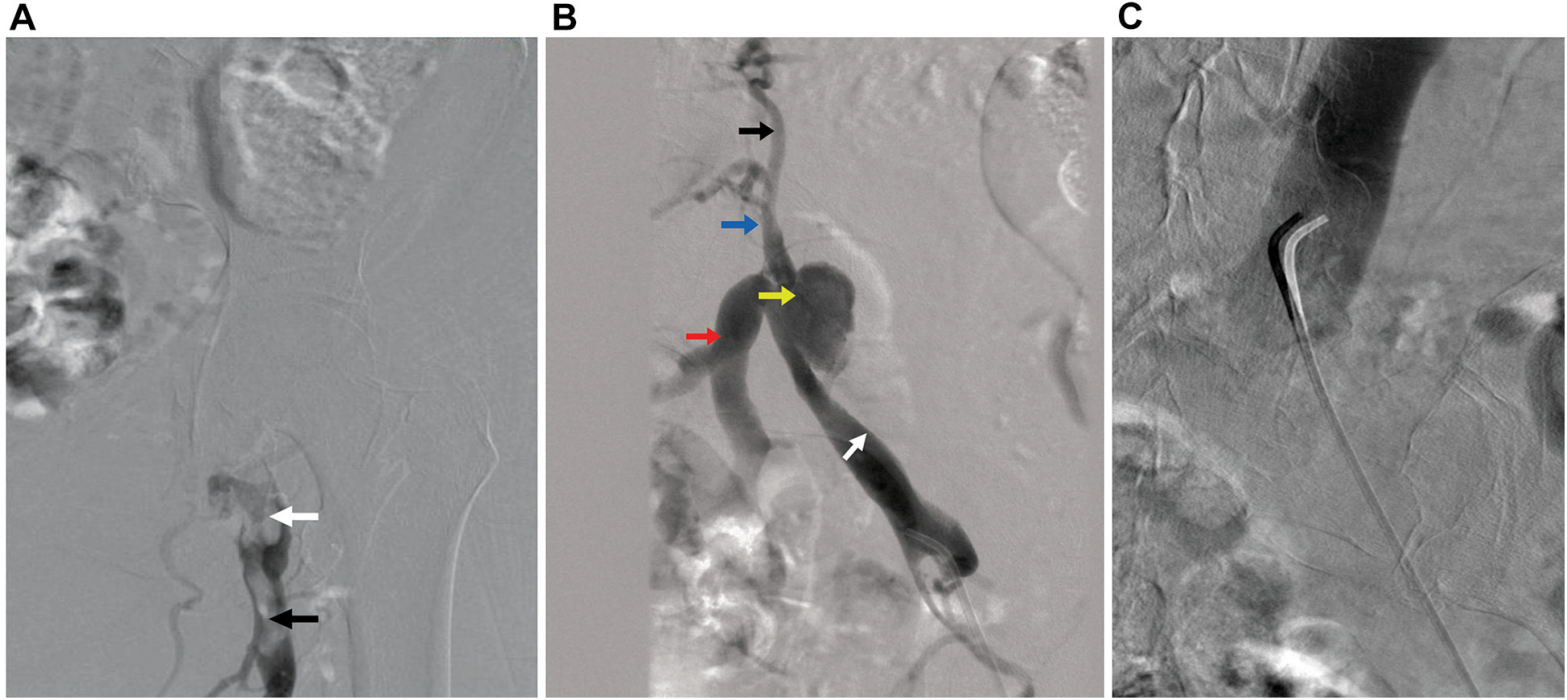
Digital subtraction angiography. **(A)** An acute occlusive thrombus at the level of the common femoral vein (white arrow) extending to the femoral vein (black arrow) is seen in the right lower extremity. **(B)** Imaging of venous outflow via a catheter at the level of the external iliac vein (white arrow) shows the venous pseudoaneurysm (yellow arrow) at the level of the internal iliac vein (red arrow). Note the occlusion of the common iliac vein (blue arrow) and opacification of collateral veins (black arrow). **(C)** Imaging of the inferior vena cava via a catheter across the venous outflow occlusion.

**Figure 2:**
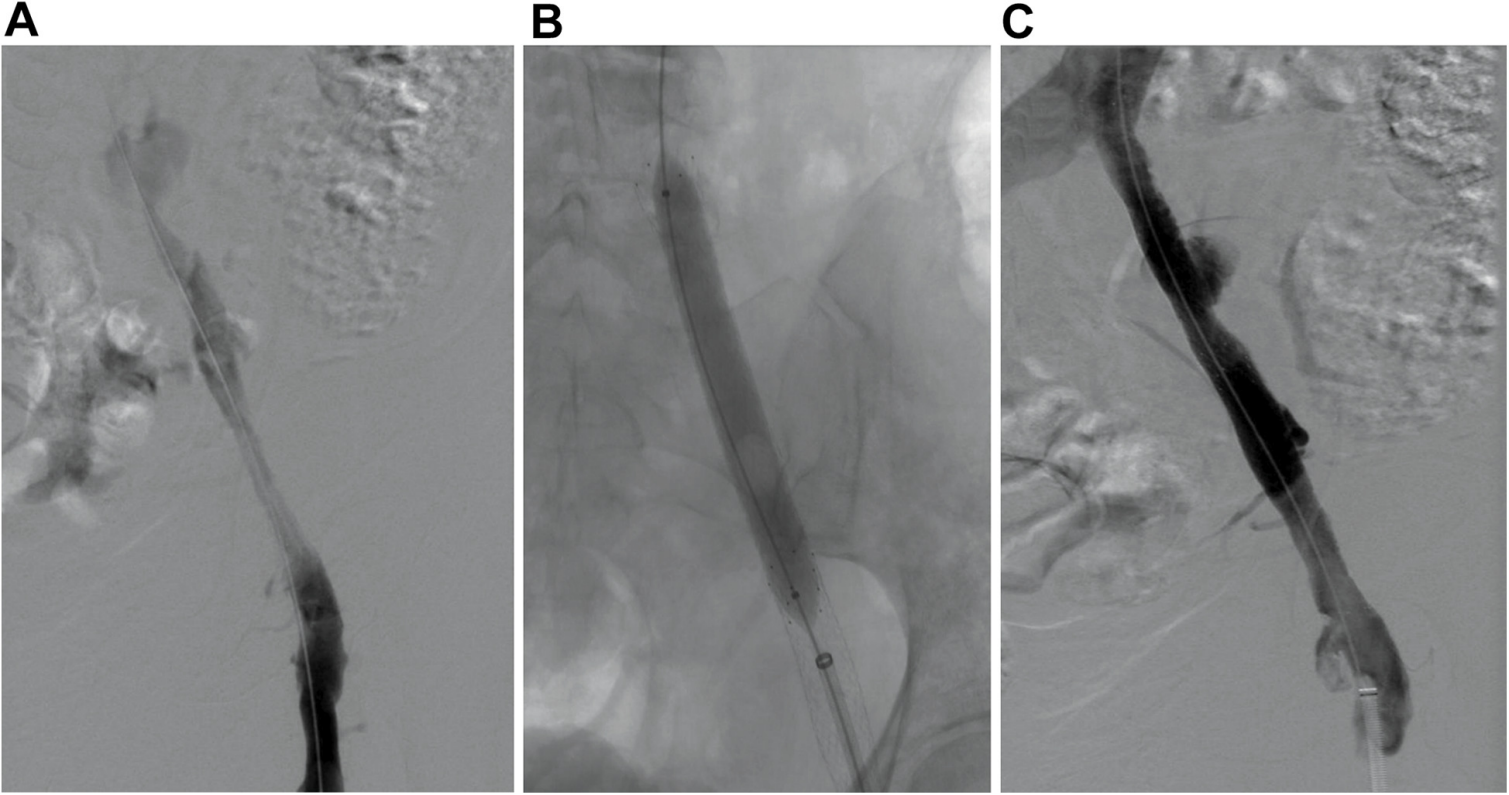
Stent deployment. **(A)** Digital subtraction angiography after mechanical thrombectomy shows prograde flow through the venous outflow. **(B)** Percutaneous transluminal angioplasty of the deployed stents. **(C)** Digital subtraction angiography after stenting shows patency of venous outflow.

**Figure 3:**
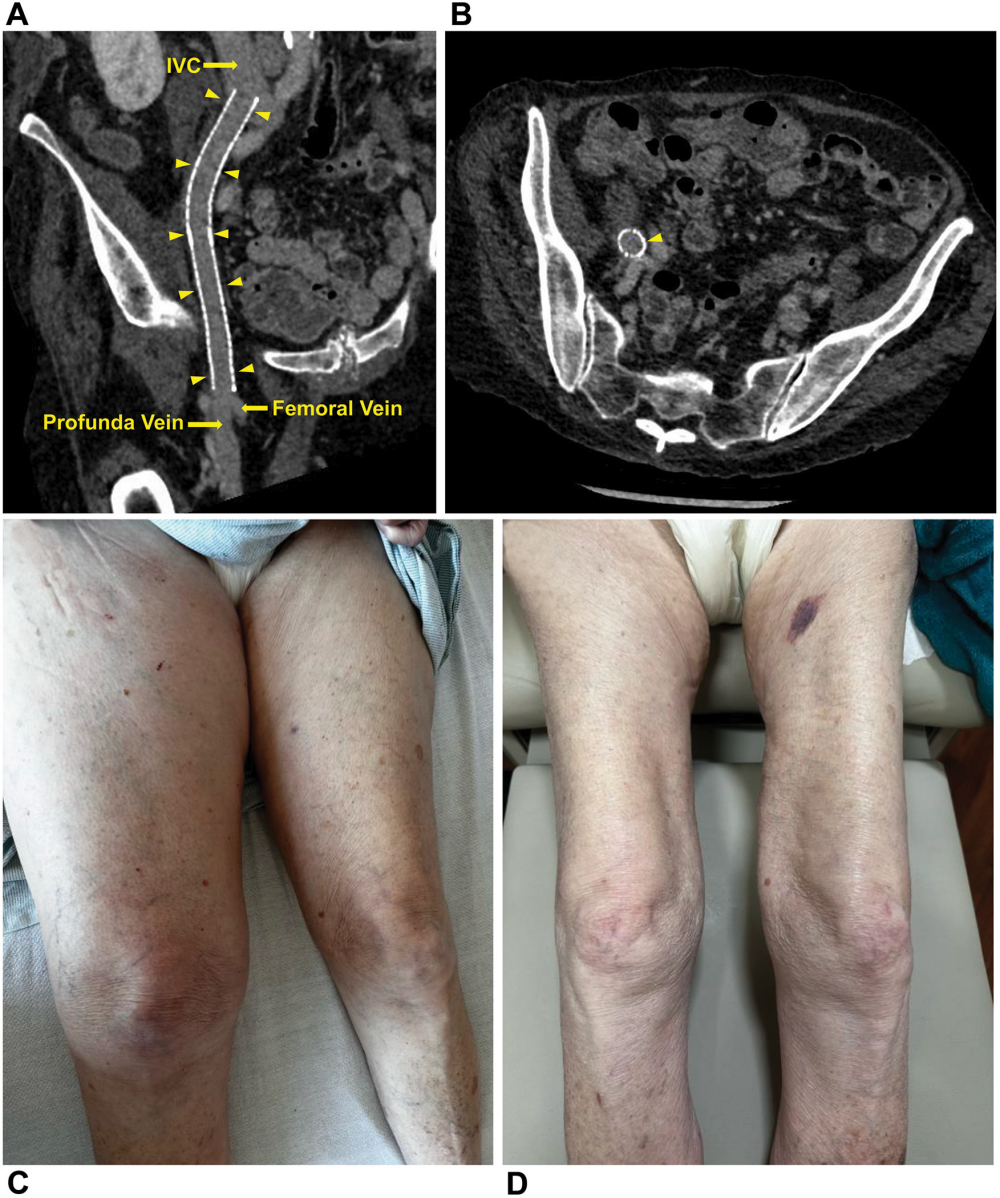
Patency confirmed, clinical improvement seen. **(A)** Longitudinal oblique view of the iliofemoral stent (arrowheads) shows the stent is patent and there is no evidence of a venous pseudoaneurysm. **(B)** Cross-sectional view of the iliofemoral stent (arrowhead) at the level of the common iliac vein bifurcation shows the stent is patent and there is no evidence of a venous pseudoaneurysm. **(C)** Right leg edema is seen at presentation. **(D)** Right leg edema has resolved at 2-month postoperative follow-up.

Post-procedure, she reported pain in the right lower extremity; vascular and neurological findings were unremarkable. The patient exhibited no chest discomfort, respiratory symptoms, or hypoxia, and was hemodynamically stable. The following day, venous ultrasound showed right proximal femoral and CFV deep vein thrombosis (DVT). Intravenous heparin was initiated and transitioned to oral apixaban before discharge. A 7-day follow-up venous ultrasound of the right lower extremity showed persistent DVT. The patient reported being medication-compliant and had mild lower extremity pain with minimal swelling. Medical treatment was continued. Seven days later, she presented with worsening right lower extremity pain, progressive swelling, and inability to ambulate. Ultrasound showed a new acute/subacute thrombus in the right external iliac vein (EIV) and the previously documented thrombosed venous segments. Intravenous heparin was initiated upon admission. Venography with intention of mechanical thrombectomy was undertaken. In the prone position, the patient underwent percutaneous catheterization of the right popliteal vein in an antegrade fashion. Venography revealed complete occlusion of the right CFV (Figure 1A; Supplementary Videos S1, S2). A 5F 65-cm catheter (GLIDECATH, Terumo Interventional Systems, Somerset, NJ) was advanced past the femoral occlusion with the aid of a 0.035-inch guidewire (Glidewire, Terumo Interventional Systems) (Figure 1B, Supplementary Video S3). Venography via the EIV indicated a patent, small vessel and an occluded common iliac vein (CIV). Venography also revealed a 3-cm pseudoaneurysm with irregular margins, suggesting partial thrombosis, at the bifurcation with the internal iliac vein. A 0.018-inch guidewire (Glide Advantage, Terumo Interventional Systems) was negotiated across the CIV occlusion to access the inferior vena cava, confirmed by venography (Figure 1C). After exchanging for a 0.035-inch guidewire (Hi-Torque Supra Core, Abbott Cardiovascular), the initial sheath was replaced with a 16F sheath after preclosing the popliteal venous access with 2 Perclose ProStyle closure devices. Through the sheath, Penumbra's Indigo Aspiration System (Alameda, Calif.) was activated several times to clear thrombi up to the inferior vena cava (Supplementary Videos S4, S5), leaving small but patent external and common iliac veins (Figures 2A,B; Supplementary Videos S6, S7). Bare-metal stents were used (10 mm diameter stent in EIV, 12 mm diameter stent telescoped to distal CIV) followed by post dilation with 10 × 80 mm and 12 × 40 mm balloons. Post-venogram results were excellent (Figure 2C; Supplementary Video S8). The sheath was removed and uncomplicated hemostasis of the access site achieved. The procedure utilized intravenous anticoagulation, maintaining an activated clotting time >250 s throughout. Warfarin was initiated. At 4-week follow-up, lower extremity pain and swelling had resolved. Venous duplex ultrasonography showed the treated veins were patent; computed tomography venography at 2-months post-operation confirmed this and showed complete resolution of the pseudoaneurysm (Figure 3). Table 1 presents a timeline of the case.

**Table 1:** Timeline.^a^

Days after TTVI	Event
Day 0	`Pain in the right groin radiating distally to the lower extremity.
	TTVI performed.
Day 1	Arterial ultrasound of lower extremities unremarkable.
	Venous ultrasound shows right proximal femoral and common femoral DVT.
	Heparin initiated.
Day 2	Patient discharged home on apixaban.
Day 7	Minimal lower-extremity swelling.
	Repeat venous ultrasound results show same as above, new acute DVT in the mid-to-distal femoral vein and popliteal vein.
	Medical treatment with apixaban continued.
Day 14	Worsening right extremity swelling and pain; patient unable to ambulate.
	Repeat ultrasound shows acute/subacute DVT in the right external iliac, in addition to the above DVTs.
	Patient admitted to hospital and started on heparin infusion.
	Mechanical thrombectomy achieved.
Day 17	Patient discharged on warfarin.
Day 47	Pain has resolved and swelling significantly reduced.
	Venous ultrasound shows patent treated veins.
Day 60	Repeat computed tomography confirms patency of treated veins and complete resolution of pseudoaneurysm.

DVT, deep vein thrombosis; TTVI, transcatheter tricuspid valve intervention.

^a^ During index hospitalization and follow-ups, the patient exhibited no chest discomfort, respiratory symptoms, or hypoxia, and was hemodynamically stable.

## 3 Discussion

The high surgical risk and operative mortality associated with isolated tricuspid valve surgery (2) has led to the advancement of TTVI, which has grown significantly over the past decade and was granted its first US Food and Drug Administration approval in February 2024 (1, 3). TTVI requires the CFV as the main access site to deliver the device through a large-caliber sheath (up to 42F) (4). Endothelial venous injury at the insertion site is a known precursor of acute thrombosis, particularly following endovascular procedures requiring a large-bore access sheath (5). Additionally, vascular closure devices, frequently used after these procedures, could play a potential pro-thrombotic role via various mechanisms, such as collagen-induced inflammatory reactions, vessel stenosis with subsequent decreased blood flow, or endothelial trauma, as seen with sutured-mediated vascular closure devices like the Perclose ProStyle used in our case (6, 7).

Our patient developed extensive DVT following large-bore sheath placement. Extensive DVT is associated with increased risk of pulmonary embolism and recurrent thrombosis and also predisposes to development of severe post-thrombotic syndrome months to years after the initial DVT (8, 9). Consequently, early invasive interventions are often entertained, particularly following anticoagulation failure or worsening symptoms as in our case (10, 11). We proceeded with percutaneous mechanical thrombectomy, a procedure proven to acutely restore venous patency. With advanced technology, associated blood loss during mechanical thrombectomy is minimal, especially with utilization of Penumbra's Indigo Aspiration System (12). This system allows a computer algorithm to optimize thrombus detection and apply continuous aspiration solely in a patent vessel with active flow. Previous data demonstrated high safety and success rates with percutaneous mechanical thrombectomy conducted in a single session, a finding validated by our own experience (12).

Other venous complications may arise from the percutaneous access required for this new TTVI procedure. In our case, we incidentally uncovered an EIV pseudoaneurysm, an unreported complication following large-sheath tracking through the iliofemoral venous segment. Although pelvic venous pseudoaneurysms due to trauma or iatrogenic causes have been described, there is currently no established consensus on the optimal treatment of these and available data are limited to case reports. Hemodynamically stable patients may be safely managed conservatively with close surveillance (13); however, the operative approach remains widely used, especially in unstable cases (14, 15). Although endovascular interventions with covered stents are typically used to treat arterial pseudoaneurysms in select cases (16, 17), their application in venous intervention has also been described. For example, Todorov et al. reported successful exclusion of an aneurysm in the external iliac vein using an endovascular approach with a covered stent graft (18). Similarly, DeWane et al. described a 35-year-old woman with a large aneurysm in the left common iliac vein that was associated with a high-flow arteriovenous fistula, which was managed using an aortic stent graft and an Amplatzer plug (Abbott Cardiovascular) in the internal iliac artery to eliminate the fistula and decompress the aneurysm (19). Additionally, uncovered bare-metal stents have been employed in both emergent and elective settings for iliofemoral venous pathology. For instance, Sofue et al. described a life-threatening iliac vein injury following blunt pelvic trauma managed with a bare-metal Wallstent (Boston Scientific, Marlborough, Mass.), where covered stents were unavailable (20). Jayaraj et al. described 3 cases of iliofemoral venous aneurysms, including an 85 × 45 mm external iliac vein aneurysm, all successfully excluded using uncovered bare-metal stents without the use of coils or grafts (21).

Similarly, in our case, we elected to use uncovered bare-metal stents, primarily to preserve internal iliac vein flow in a patient who already had compromised venous outflow. On the arterial side, severe complications that can result in rapid demise, such as complete iliac artery disruption, have been reported post-percutaneous intervention (22). Such disruption could occur on the venous side with large-bore sheaths, and the operator should be equipped to treat them promptly.

Appropriate venous stent sizing is crucial. We initially deployed a 10-mm stent in the distal CFV and subsequently deployed a 12 mm stent in the EIV. These stent sizes had been validated previously based on the absolute cross-sectional area of the vein (23). Although the natural history of iliac venous pseudoaneurysm is unknown, we postulated an unlikely risk of enlargement and rupture given a low-pressure environment. The metallic stent deployed across the lesion was sufficient to contain the pseudoaneurysm, which was not visible at the 2-month follow-up.

In the pivotal TRISCEND II (EVOQUE Transcatheter Tricuspid Valve Replacement: Pivotal Clinical Investigation of Safety and Clinical Efficacy Using a Novel Device) trial (24), the rate of major access site and vascular complications in the valve replacement group was 3.1% (8 of 259 patients). Specific vascular complications such as DVT or venous pseudoaneurysm were not individually reported; our case adds valuable real-world insight into these underreported events and emphasizes the need for awareness and proactive management following TTVI. A study with a larger sample size will likely highlight the incidence of such complications in the near future. However, transcatheter intervention remains vital in such cases.

## 4 Conclusion

It is important to implement a surveillance strategy, e.g., duplex ultrasonography, immediately post-TTVI to rule out acute thrombosis of the venous access site and other complications associated with a large sheath, such as venous pseudoaneurysm.

## Data Availability

The original contributions presented in the study are included in the article/Supplementary Material, further inquiries can be directed to the corresponding author.
